# Doxycycline increases neurogenesis and reduces microglia in the adult hippocampus

**DOI:** 10.3389/fnins.2013.00131

**Published:** 2013-07-25

**Authors:** Sebastien Sultan, Elias Gebara, Nicolas Toni

**Affiliations:** Department of Fundamental Neurosciences, University of LausanneLausanne, Switzerland

**Keywords:** dentate gyrus, hippocampus, adult neurogenesis, doxycycline, tetracycline, gene expression regulation

## Abstract

Adult hippocampal neurogenesis results in the continuous formation of new neurons and is a process of brain plasticity involved in learning and memory. Although inducible-reversible transgenic mouse models are increasingly being used to investigate adult neurogenesis, transgene control requires the administration of an activator, doxycycline (Dox), with unknown effects on adult neurogenesis. Here, we tested the effect of Dox administration on adult neurogenesis *in vivo*. We found that 4 weeks of Dox treatment at doses commonly used for gene expression control, resulted in increased neurogenesis. Furthermore, the dendrites of new neurons displayed increased spine density. Concomitantly, Iba1-expressing microglia was reduced by Dox treatment. These results indicate that Dox treatment may interfere with parameters of relevance for the use of inducible transgenic mice in studies of adult neurogenesis or brain inflammation.

## Introduction

Adult neurogenesis occurs mainly in two discrete areas: the olfactory bulb and the dentate gyrus (DG) of the hippocampus (Altman, 1969). The process of adult neurogenesis consists in several steps: First, the division of adult neural stem cells, residing in the subgranular zone (SGZ) of the DG (Gage, 2000). Adult neural stem cells display a radial glia like (RGL) morphology, characterized by a unique radial process extending through the granule cell layer and branching into the molecular layer and by the expression of several markers such as the astrocytic glial fibrillary acidic protein (GFAP) and nestin (Huttmann et al., 2003; Ehninger and Kempermann, 2008). RGL cells divide asymmetrically to self-renew and yield highly proliferative transit-amplifying progenitors (TAPs), which do not have a radial morphology and express nestin and the T-box brain gene 2 (Tbr2), but not GFAP. TAPs give rise to immature neurons, which express the immature neuronal marker doublecortin (DCX) and differentiate into mature, NeuN-expressing neurons (Gage, 2000; Ma et al., 2009; Yao et al., 2012). During their maturation, young neurons project their dendrites into the molecular layer, form dendritic spines, which receive synaptic inputs from perforant path afferences from the entorhinal cortex (Gage, 2000; Van Praag et al., 2002; Laplagne et al., 2006; Toni et al., 2007; Toni and Sultan, 2011). They also project their axons to the hippocampal CA3 area and establish synaptic connections with postsynaptic inhibitory interneurons and excitatory pyramidal neurons (Toni et al., 2008). After 8 weeks, new neurons are morphologically and functionally indistinguishable from neighboring neurons and are completely integrated into the hippocampal network (Laplagne et al., 2006; Ge et al., 2008).

The mechanisms regulating adult neurogenesis are highly relevant for our understanding of brain plasticity and for the potential use of these cells as therapeutic targets. Indeed, although their role remains unclear, increasing evidence suggests that new neurons are involved in mechanisms of learning and memory (Van Praag et al., 1999, 2002; Saxe et al., 2006, 2007; Dupret et al., 2007; Trouche et al., 2009; Massa et al., 2011; Gu et al., 2012; Shors et al., 2012; Tronel et al., 2012) as well as in depression and mood control (Santarelli et al., 2003; Samuels and Hen, 2011). However, one of the great difficulties in studying adult neurogenesis *in vivo*, is that the mechanisms regulating neuronal proliferation, differentiation and survival also play a role during brain development and therefore, their manipulation leads to prenatal death or morbidity. This experimental caveat can be circumvented by the use of inducible transgene expression, such as the tetracycline regulatory system, controlled with the administration of doxycycline (Dox) (Gossen and Bujard, 1992; Zhou et al., 2006; Chow et al., 2012). This elegant system enables the bi-directional and non-invasive regulation of gene expression. Furthermore, by controlling the timing of the genetic manipulation, genes necessary for cell survival can be manipulated without affecting brain development. However, although inducible transgenic mouse models are increasingly being used for studies of adult neurogenesis, the effect of Dox on adult neurogenesis is unknown. Dox is a synthetic antibiotic of the tetracycline inhibitors group, with reported effects on pain, inflammation and neuroprotection (Clark et al., 1997; Cho et al., 2009; Jantzie and Todd, 2010; Yoon et al., 2012). Thus, the use of Dox to control gene expression may potentially bias phenotypic analysis of adult neurogenesis with the introduction of confounding factors.

The aim of the present study was to examine the potential effects of Dox on adult neurogenesis *in vivo*. We have used a *per os* administration route, which corresponds to the main experimental protocol for the control of transgene expression. Using immunohistochemistry and viral-mediated labeling, we have analyzed the effect of Dox on RGL cells, TAPs and on the differentiation, survival and synaptic maturation of new neurons, as well as on microglia.

## Materials and methods

### Ethics statement

This study was carried out in strict accordance with the recommendations in the Guidance for the Care and Use of Laboratory Animals of the National Institutes of Health. All experimental protocols were approved by the Swiss animal experimentation authorities (Service de la consommation et des affaires vétérinaires, Chemin des Boveresses 155, 1066 Epalinges, Switzerland, permit number: 2301). Every effort was made to minimize the number of animals used and their suffering.

### Experimental animals

Animals used for the study were adult males of 6 weeks of age at the beginning of the experiment. All animals were housed in standard cages under a 12-h light/dark cycle and temperature-controlled (22°C) conditions. Food and water were available *ad libitum*. C57Bl/6 mice were purchased from Janvier (le Genest Saint Isle, France), GFAP-GFP mice were a kind gift from the laboratory of Helmut Kettenmann (Max-Delbruck center, Berlin, Germany) (Nolte et al., 2001). They express the green fluorescent protein (GFP) under the control of the astrocyte-specific human GFAP promoter. Nestin-GFP mice were a kind gift from the laboratory of K. Mori (PRESTO, Kyoto, Japan) (Yamaguchi et al., 2000). They express GFP under the control of the stem cell-specific promoter nestin. Food pellets containing 40-ppm of Dox were purchased from Harlan Laboratory (WI, USA). Control mice were fed with the same food pellets without Dox, purchased from the same source.

### BrdU administration

Bromodeoxyuridine (BrdU, Sigma-Aldrich, Buchs, Switzerland) was injected intraperitoneally at doses of 100 mg/kg in saline, 3 times at 2-h intervals. Mice were then sacrificed either 2 h after the last injection, to examine cell proliferation (Mandyam et al., 2007; Taupin, 2007; Yang et al., 2011; Gao and Chen, 2013) or 30 days after injections, to examine newborn cells survival (Taupin, 2007).

### Retrovirus-mediated labeling

We used a retroviral vector derived from the Moloney murine leukemia virus (MoMuLv) containing a GFP-expression cassette under the control of the cytomegalovirus early enhancer and chicken beta-actin promoter (cag) (Zhao et al., 2006). The final virus titer was 10E8 pfu/ml, as measured by GFP–expressing colony formation on 293T cells. Mice were anesthetized with a mixture of 90 mg/kg ketamine and 4.5 mg/kg xylazine (i.p.) and then placed in a stereotaxic instrument (Narishige Scientific Instruments, Tokyo, Japan). 1.5 μl of virus was injected bilaterally at the following coordinates from the Bregma: anteroposterior −2 mm, lateral 1.75 mm and dorsoventral −2.25 mm. GFP signal was amplified by immunohistochemistry using chicken anti-GFP IgY (AnaSpec Inc. CA 94555, 1:1000) and dylight 488 goat anti-chicken IgY (Jackson ImmunoResearch Europe ltd., Suffolk, United Kingdom; 1:250).

### Tissue collection and preparation

Mice were deeply anesthetized with a lethal dose of pentobarbital (10 mL/kg, Sigma-Aldrich, Buchs, Switzerland) and perfusion-fixed with 50 ml of 0.9% saline immediately followed by 100 mL of 4% paraformaldehyde (Sigma-Aldrich, Switzerland) dissolved in phosphate buffer saline (PBS 0.1 M, pH 7.4). Brains were then dissected, post-fixed overnight at 4°C, cryoprotected 24 h in 30% sucrose and rapidly frozen. Coronal frozen sections of a thickness of 40 μm were cut with a microtome-cryostat (Leica MC 3050S) and slices were stored in cryoprotectant (30% ethylene glycol and 25% glycerin in 1X PBS) at −20°C until processing for immunostaining, as described previously (Thuret et al., 2009).

### Immunohistochemistry

Sections were washed 3 times in PBS 0.1M. BrdU detection required formic acid pretreatment (formamide 50% in 2× SSC buffer; 2× SSC is 0.3 M NaCl, and 0.03 M sodium citrate, pH 7.0) at 65°c for 2 h followed by DNA denaturation for 30 min in 2 M HCl at 37°C and rinsed in 0.1 M borate buffer pH 8.5 for 10 min. Nonspecific binding was blocked with 0.25% Triton-X100 and 15% normal serum [normal goat serum (Gibco, 16210-064) or normal donkey serum (Sigma Aldrich, D-9663), depending on the secondary antibody] in PBS 0.1 M. Slices were then incubated 48 h at 4°C with the primary antibodies described below. Then sections were incubated for 2 h with the corresponding secondary antibodies in PBS 0.1 M. 4,6 diamidino-2-phenylindole (DAPI) was used to reveal nuclei.

Primary antibodies used for immunohistochemistry were as follows: rabbit anti-Ki-67 (1:200, Abcam, ab15580), goat anti-DCX (1:500, Santa Cruz biotechnology, sc-8066), rabbit anti-Tbr2 (1:200, Abcam, ab23345), mouse monoclonal anti-BrdU (1:250, Chemicon International, Dietikon, Switzerland), mouse anti-NeuN (Chemicon international 1:1000), goat anti-Iba1 (1:200, Abcam, ab5076), rabbit anti-GFAP (1:500, Invitrogen, 180063).

Secondary antibodies were used as follows: goat anti-mouse Alexa-594 (1:250, Invitrogen), goat anti-rabbit Alexa-594 (1:250, Invitrogen), goat anti-rabbit Alexa-488 (1:250, Invitrogen), donkey anti-goat Alexa-555 (1:250, Invitrogen).

### Image analysis

All images were acquired using a confocal microscope (Zeiss LSM 710 Quasar Carl Zeiss, Oberkochen, Germany). The total numbers of immunoreactive cells throughout the entire granule cell layer were estimated using stereological sampling, as previously described (Thuret et al., 2009), between −1.3 and −2.9 mm from the Bregma. However, no guard zones were used, which may lead to possible bias in the counting of cells at the edge of each section, spread across control and Dox groups. For each animal, a 1-in-6 series of sections was stained with the nucleus marker DAPI and used to measure the volume of the granule cell layer. The granule cell area was traced using Axiovision (Zeiss, Germany) software and the granule cell volume was determined by multiplying the traced granule cell layer area by the thickness of the corresponding section and the distance between the sections sampled (240 μm). For all mice analyzed in this study, no difference was found between Dox-treated and control animals. All cells were counted blind with regard to the mouse status. Cells were counted in the entire thickness of the sections in a 1-in-6 series of section (240 μm apart) with a 40× objective. The number of immunolabeled cells was then related to granule cell layer sectional volume and multiplied by the reference volume to estimate the total number of immunolabeled cells. Cells expressing BrdU, Ki-67, DCX or Tbr2 were counted in the granule cell layer, whereas cells expressing Iba1 and GFAP (Figure 6) were counted in the whole DG.

BrdU colocalization with the neuronal marker NeuN was analyzed by confocal microscopy and was confirmed on single optical sections, for 50–60 cells per animal. The proportion of double-labeled cells was then obtained for each animal and then averaged for each group. DCX-expressing cells were counted on confocal stack images using the colocalization with the nuclear stain DAPI and/or the presence of processes as visual landmarks for their identification. This approach may lead to a slight underestimation of DCX-expressing cell numbers. Spine density was assessed as previously described (Krzisch et al., 2013). Dendrites were imaged with confocal microscopy in the second third of the molecular layer and their length as well as spine density (number of spines divided by dendritic length) was measured using image J software, for 40–50 neurons per group.

Spine morphology was classified in three groups based on the maximal diameter of the spine head, as measured on maximal projections with Image J software: Filopodia <0.25μm, thin spines 0.25–0.45 μm and mushroom spines >0.45 μm. The percentage of each type of dendritic spine was then expressed by neuron and averaged for each mouse (25–30 neurons per group, 800 spines per group).

### Cell culture

Adult neural progenitor cells (NPC) expressing the red fluorescent protein (RFP) are a kind gift from the laboratory of Fred Gage (Salk Institute, San Diego, USA). They were originally isolated from the DG of adult Fisher 344 rats and cultured as previously described (Palmer et al., 1997).

Microglia and astrocyte primary culture were purified from postnatal day 2 rats. Cerebral cortices were mechanically triturated for homogenization and seeded onto poly-D-lysine coated 75 cm^2^ flasks in Dulbecco's Modified Eagle Medium (DMEM) glutamax (Invitrogen, USA), 10% normal calf serum with penicillin/streptomycin (Invitrogen, USA). Cells were grown for 5–7 days in a humidified 5% CO2 incubator at 37°C. At confluence, flasks were shaken at 250 rpm on an orbital shaker for 2 h to separate microglia from astrocytes. Detached microglia were seeded in poly-D-lysine coated 6-well microplates in culture medium supplemented with 30% astrocyte conditioned medium.

All three cell types were cultured separately and, one day after plating, were treated with Dox or vehicle (PBS). Dox was purchased from Sigma-Aldrich (St Louis, MO, USA) and dissolved in PBS to prepare a stock solution of 10 mg/ml. The stock solution was stored at −20°C. Upon use, the stock solution was diluted 10 times in PBS and 1 μ l of the solution was added daily to the culture medium, at a concentration of 1 μg/ml. This regimen of Dox treatment is commonly used for the induction of tetracycline-dependent gene expression in cell culture (Stegmeier et al., 2005; Richter et al., 2013).

After the treatment, cells were fixed and mounted for cell quantification. The number of Iba1+, RFP+, and GFAP+ cells was counted in twelve randomly selected fields per condition (three culture wells per groups, four fields per culture well) on confocal micrographs. The number of cells was then divided by the surface area of the selected fields, to obtain cell density. The density was then averaged between the four fields to obtain the average density per culture well.

### Statistical analysis

Hypothesis testing was two-tailed. All analyses were performed using JMP10 software. First, Shapiro-Wilk tests were performed on each group of data to test for distribution normality. The distribution was normal for all data. For two-sample comparisons, the equality of variances of the groups was tested and the adequate unpaired *t*-test was used. For the analysis of dendritic spine morphology (Figure 5C), parametric tests were used (two-way ANOVA followed by a *post-hoc* Student's *t*-test). Data are presented as mean ± SEM.

## Results

### Dox exposure increased cell proliferation

**Figure 1:**
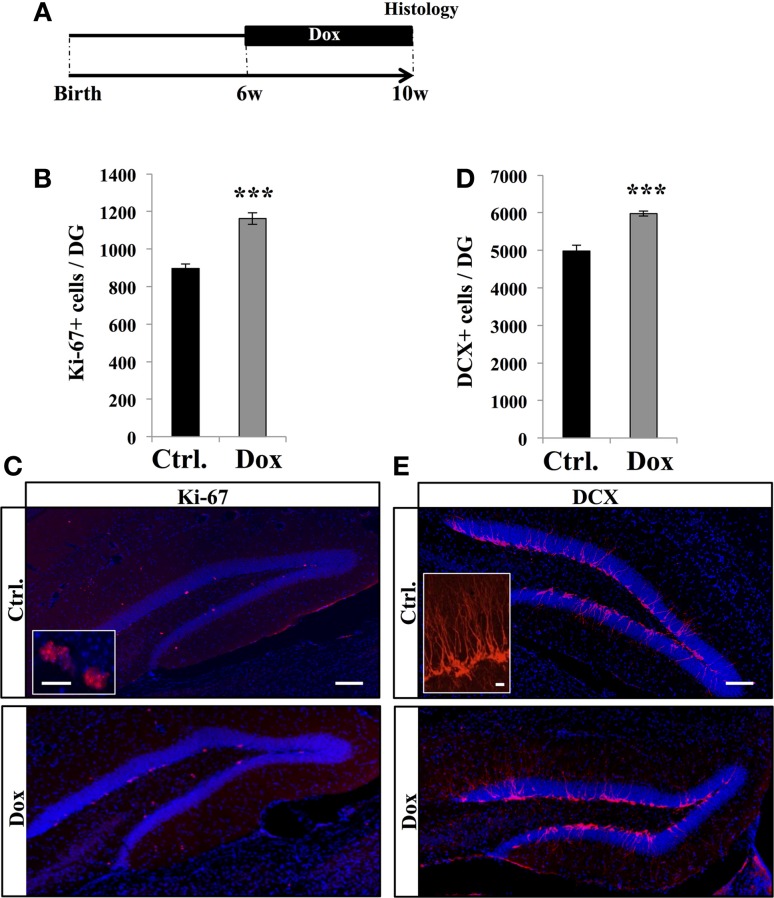
**Dox effect on cell proliferation. (A)** Experimental timeline: C57Bl/6 mice were treated with control food (Ctrl.) or food containing Dox for 4 weeks and sacrificed at 10 weeks of age. **(B)** Histogram showing the number of Ki-67–expressing cells in the dentate gyrus. Animals: *n* = 5 per group. **(C)** Confocal micrographs of hippocampal sections immunostained with Ki-67 in control animals (upper panel), and treated animals (lower panel). Inset: Higher magnification confocal micrograph of a Ki-67-expressing cell. **(D)** Histogram showing the number of doublecortin (DCX)-immunolabeled cells in the dentate gyrus. Animals: *n* = 5 per group. **(E)** Confocal micrographs of hippocampal sections immunostained for DCX from control animals (upper panel), and treated animals (lower panel). Inset: Higher magnification confocal micrograph of a DCX-immunolabeled group of cells. Blue: Dapi staining. Scale bars: 100 μm, insets 10 μm, ^***^p < 0.001 bilateral Student's *t*-test. Each value represents the mean ± SEM.

To examine the effect of Dox treatment on cell proliferation, 6 weeks old C57Bl/6 mice were fed for 4 weeks with unlimited supply of food pellets containing either 40 ppm of Dox or no Dox (Figure 1A). At the end of the treatment, mice were sacrificed and brain slices were immunostained for the proliferation marker Ki-67 (Kee et al., 2002). Treated animals showed a higher number of Ki-67 labeled cells in the granule cell layer of the DG than control mice (Figures 1B,C, Student's *t*- test, *p* < 0.001) suggesting an increased number of cells in the cell cycle.

We next examined the effect of Dox treatment on immature neurons expressing DCX. Dox treatment significantly increased the number of immature neurons (Figures 1D,E, Student's *t*-test, *p* < 0.001). Thus, Dox treatment enhanced proliferation in the adult hippocampus and increased the number of immature neurons. To test whether the increased number of Ki-67- and DCX-expressing cells could be caused by a change in hippocampal volume upon Dox treatment, we measured the volume of the granule cell layer of all mice. We did not detect a difference between treated and untreated animals (0.16 ± 0.05 mm^3^ vs. 0.15 ± 0.01 mm^3^, respectively, *n* = 5 and Student's *t*-test, *p* = 0.7), indicating that the increased number of Ki-67 and DCX-immunolabeled cells reflected an increase in proliferation.

**Figure 2:**
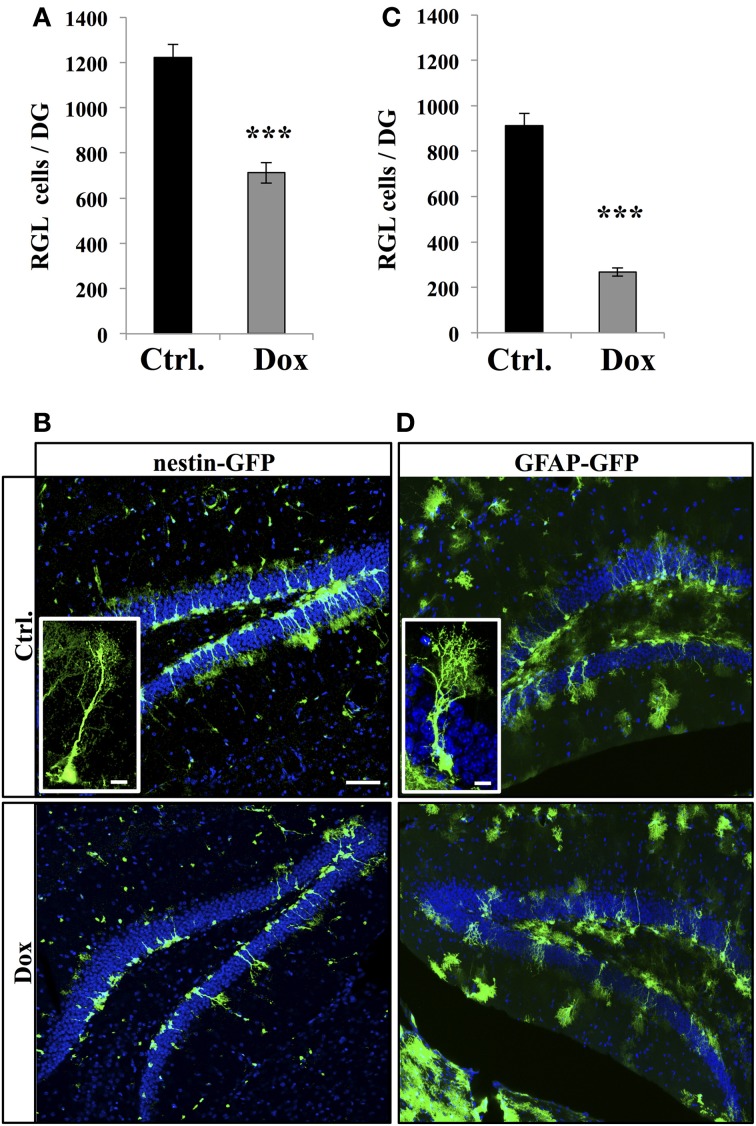
**Dox effect on the number of RGL cells in the dentate gyrus. (A)** Histogram showing the number of RGL cells in the dentate gyrus of nestin-GFP mice exposed to control (Ctrl.) or Dox treatment. **(B)** Confocal micrographs of hippocampal sections from control (upper panel) and treated animals (lower panel). Inset: higher magnification confocal micrograph of a RGL cell. **(C)** Histogram showing the number of RGL cells in the dentate gyrus of GFAP-GFP mice exposed to control (Ctrl.) or Dox treatment. **(D)** Confocal micrographs of hippocampal sections from control (upper panel) and treated animals (lower panel). Inset: higher magnification confocal micrograph of a RGL cell. Blue: Dapi staining. Animals: *n* = 4 per group, scale bars: 100 μm, insets 10 μm, ^***^p < 0.001 bilateral Student's *t*-test. Each value represents the mean ± SEM.

We then investigated the effect of Dox on the two main types of proliferative cells: RGL cells and TAPs. RGL cells were identified using nestin-GFP (Figures 2A,B) and GFAP-GFP (Figures 2C,D) mice, which are commonly used mouse models in studies of adult neurogenesis (Huttmann et al., 2003; Mignone et al., 2004; Beckervordersandforth et al., 2010). In both mouse lines, RGL cells are readily identifiable by their specific morphology (Kriegstein and Alvarez-Buylla, 2009), consisting of a nucleus in the SGZ and a large process extending through the granule cell layer and branching into the proximal molecular layer (Figures 2B,D, insets). With immunostaining, we confirmed that these cells expressed nestin, GFAP, and sox-2 (data not shown). Surprisingly, Dox treatment significantly reduced the number of RGL cells in both the nestin-GFP mice (Figure 2A, Student's *t*-test, *p* < 0.001) and the GFAP-GFP mice (Figure 2C, Student's *t*-test, *p* < 0.001).

**Figure 3:**
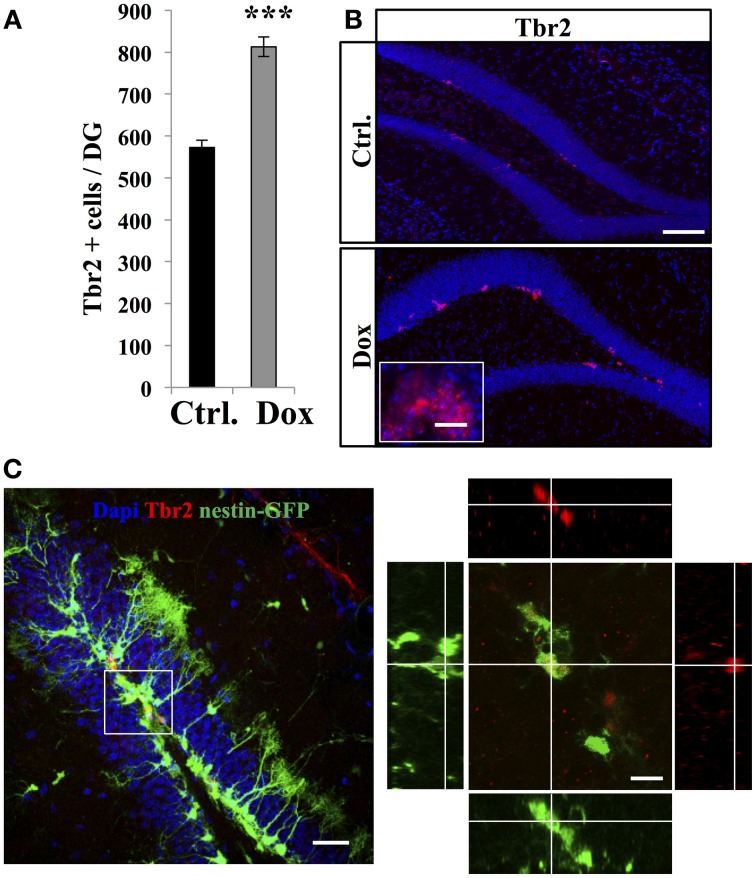
**Dox effect on the number of progenitors. (A)** Histogram showing the number of Tbr2-expressing cells in the dentate gyrus of nestin-GFP mice exposed to control food (Ctrl.) or Dox treatment. **(B)** Confocal micrographs of hippocampal sections immunostained for Tbr2 from control (upper panel) and treated animals (lower panel) scale bars: 100 μm, inset 10 μm. **(C)** Left panel: Confocal micrograph of a hippocampal section (maximum projection) from a nestin-GFP mouse immunostained for Tbr2 (red) scale bar: 50 μm Right panel: Orthogonal projection of a Tbr2-immunolabeled cell also expressing GFP. Scale bar: 15 μm. Blue: Dapi staining. Animals: *n* = 4 per group, ^***^p < 0.001 bilateral Student's *t*-test. Each value represents the mean ± SEM.

TAPs were identified using immunostaining against Tbr2, (Hodge et al., 2008, Figure 3). In untreated nestin-GFP mice, 94.5 ± 1% of TbR2 + cells co-labeled with GFP (*n* = 100 Tbr2+ cells), but 93.1 ± 2% of them did not have RGL morphology (Figure 3C). Dox treatment significantly increased the number of Tbr2-expressing cells (Figure 3A, Student's *t*-test, *p* < 0.001). Here too, we did not detect any difference in the volume of the granule cell layer between treated and control animals in both nestin-GFP treated and untreated animals (1.4 ± 0.09 mm^3^ vs. 1.51 ± 0.06 mm^3^, respectively, *n* = 4 and Student's *t*-test, *p* = 0.63) and treated and untreated GFAP-GFP mice (1.43 ± 0.04 mm^3^ vs. 1.43 ± 0.01 mm^3^, respectively, *n* = 4 and Student's *t*-test, *p* = 0.97).

Thus, Dox treatment increased the total number of the highly-proliferative progenitors, but decreased the number of the quiescent, slowly-proliferative RGL cells.

### Dox treatment increased adult neurogenesis

**Figure 4:**
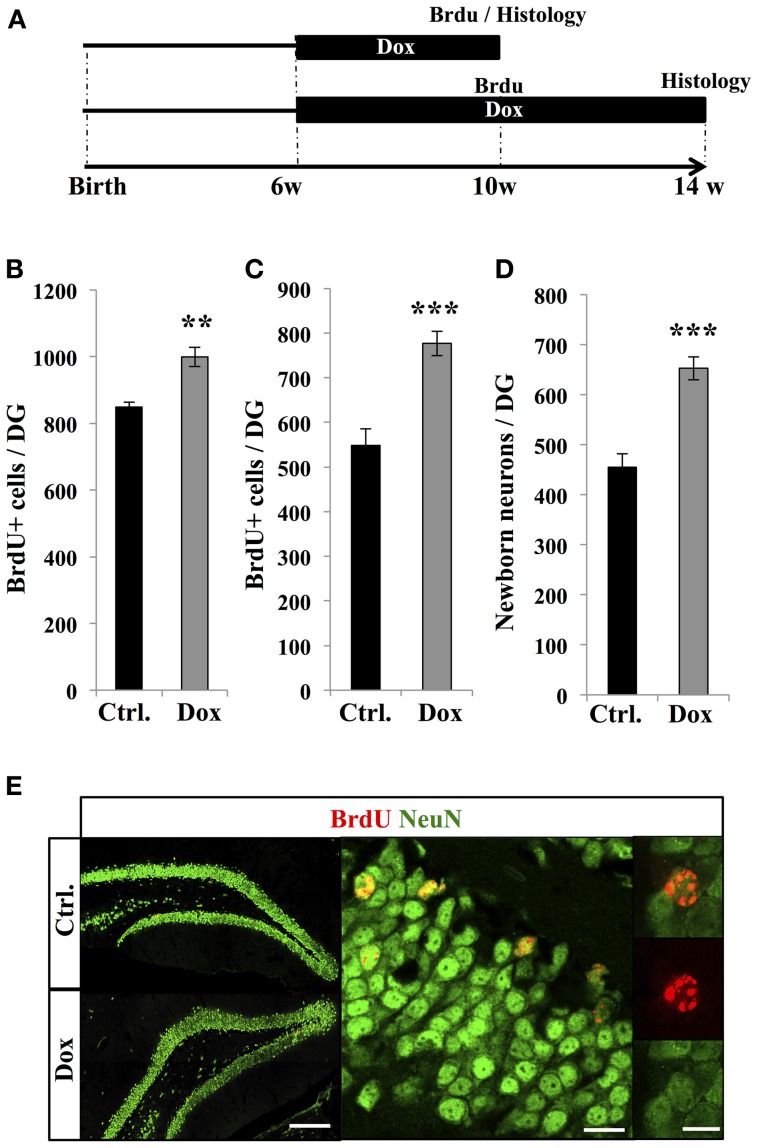
**Effect of Dox on the number of new neurons. (A)** Experimental timeline: 6 weeks old C57Bl/6 mice were treated with control food (Ctrl.) or food containing Dox for 4 weeks and received BrdU injections. The first group of mice was euthanized 2 h after the BrdU injections to assess the number of proliferative BrdU+ cells. The second group received another 4 weeks of Dox treatment, after which they were euthanized, to measure the number of surviving newborn cells. **(B)** and **(C)** Histogram showing the number of BrdU-expressing cells in the dentate gyrus of animals exposed to control (Ctrl.) or Dox food, the day of injection **(B)** or 4 weeks after BrdU injections **(C)**. Animals: *n* = 5 per group. **(D)** Histogram showing the quantification of cells expressing NeuN relative to the number of BrdU-positive cells, 4 weeks after the last BrdU injection. **(E)** Left: confocal micrographs of hippocampal sections immunostained for BrdU (red) and NeuN (green) in control animals (upper panel) and treated animals (lower panel) scale bar: 100 μm. Right: Higher magnification confocal micrographs of a BrdU-NeuN expressing cells. Scale bar: 10 μm, inset 10 μm. Animals: *n* = 3 per group, 48–69 cells per animal. ^**^p < 0.01, ^***^p < 0.001 bilateral Student's *t*-test. Each value represents the mean ± SEM.

To examine the effect of Dox treatment on the fate of newborn cells, two groups of adult C57Bl/6 mice were treated with Dox during 4 weeks, after which they received three injections of BrdU (intraperitoneal, 1.0 mg/kg in saline at 2 h intervals). One group of mice was sacrificed 2 h after the last injection, while the second group received another 4 weeks of Dox treatment and was analyzed thereafter to assess neuronal differentiation (Figure 4A). The first group showed a higher number of BrdU-expressing cells in the DG as compared to control-treated mice (Figure 4B, Student's *t*-test, *p* < 0.01), supporting our previous observation of increased cell proliferation with Dox treatment (Figure 1). Similarly, in the second group (4 weeks after the last BrdU injection), the number of BrdU-expressing cells was higher in Dox-treated mice than in control mice (Figure 4C, Student's *t*-test *p* < 0.001). Finally, we assessed the neuronal differentiation of BrdU-labeled cells into neuronal lineage, by co-labeling for BrdU and the neuron-specific marker, NeuN (Figure 4E). In control mice, neurons accounted for 83 ± 0.2% of the surviving BrdU-positive cells as compared to 84 ± 0.3% in Dox-treated mice (Student's *t*-test *p* > 0.05), indicating that differentiation was not affected by Dox treatment. All together, these results indicate that Dox-treated mice generate 43.4 ± 0.4% more neurons than non-treated mice (Figure 4D, Student's *t*-test, *p* < 0.001).

### Dox treatment increased spine density on newborn neurons

**Figure 5:**
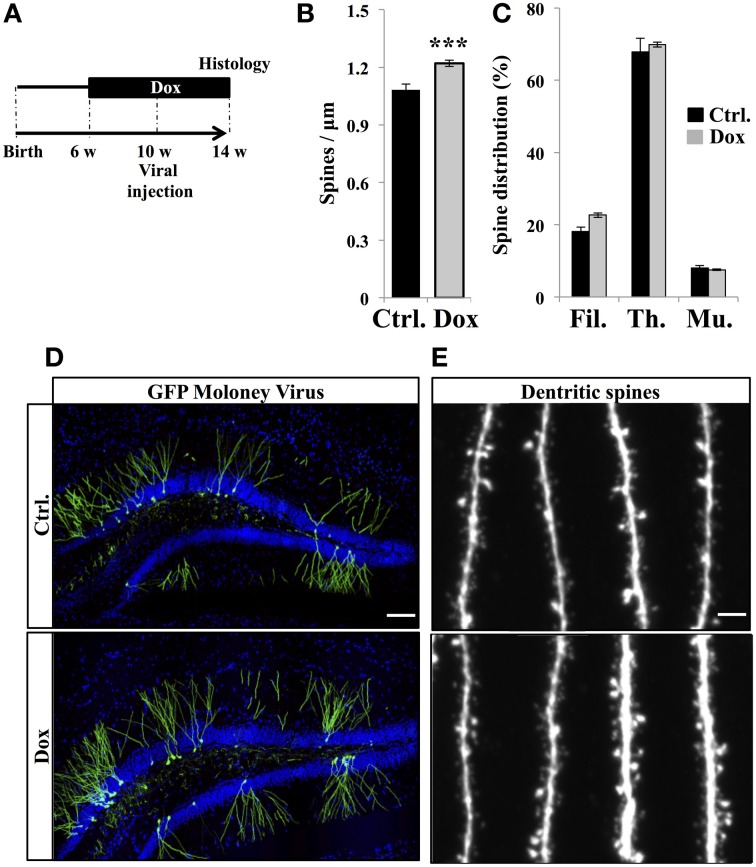
**Effect of Dox on the spine density of newborn neurons. (A)** Experimental timeline: C57Bl/6 mice were treated with control food (Ctrl.) or food containing Dox for 4 weeks before viral injection. Dox treatment was continued for 4 weeks after viral injection. **(B)** Histogram showing the spine density on newborn neurons from the two experimental conditions, *n* = 48–54 neurons per group, ^***^p < 0.001 bilateral Student's *t*-test. **(C)** Histogram showing the percentage of filopodia (Fil.), thin spines (Th.), and mushroom spines (Mu.) on newborn neurons from control-treated animals (black) and Dox-treated animals (gray) *n* = 838–895 spines per group. **(D)** Confocal micrographs of hippocampal sections of retrovirally-injected mice from control animals (upper panel) and treated animals (lower panel) Blue: Dapi staining, scale bar: 100 μm. **(E)** Confocal micrographs of spiny dendrites from control animals (upper panel) and treated animals (lower panel) scale bars: 10 μm. Each value represents the mean ± SEM.

The final stage of neurogenesis consists in the integration of the newly-formed neurons into the hippocampal excitatory circuitry and is commonly assessed by measuring dendritic spine density. To this aim, new neurons were identified by viral-mediated gene transfer, with the use of a MoMuLV containing the expression cassette for GFP. C57Bl/6 mice were treated with Dox for 4 weeks and then stereotaxically injected with GFP-retrovirus, followed by 4 weeks of Dox treatment (Figure 5A). Dendritic spines were analyzed in the middle-third of the molecular layer, where inputs arise mainly from the entorhinal cortex. At 30 days post-virus infection, Dox treatment increased spine density (Figures 5B,D,E, Student's *t*-test, *p* < 0.001). Dendritic spine diameter increases with neuronal maturation (Zhao et al., 2006; Toni et al., 2007) and reflects synaptic strength (Murthy et al., 2001). To examine the effect of Dox on dendritic spine maturation, we classified dendritic spines in three categories, based on the maximal diameter of the spine head: filopodia spines <0.25 μm, thin spines 0.25–0.45 μm and mushroom spines >0.45 μm. Dox treatment did not change the proportion of filopodia, thin and mushroom spines [Figure 5C, two way ANOVA *F*_(2, 108)_ = 1.1, *p* = 0.33], indicating that this treatment increased spine density but did not affect dendritic spine morphology.

### Dox reduced microglia but not astroglia *in vivo* and *in vitro*

**Figure 6:**
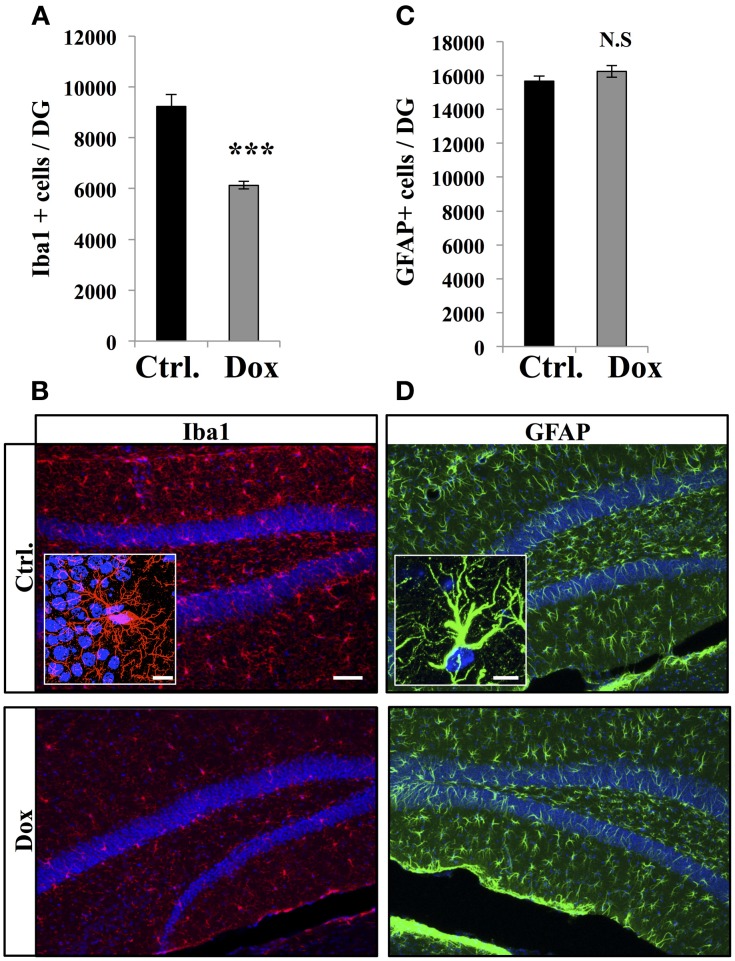
**Effect of Dox on the number of microglia and astrocytes in the dentate gyrus. (A)** Histogram showing the number of Iba1-expressing cells in the dentate gyrus of mice exposed to control food (Ctrl.) or Dox treatment. Animals: *n* = 5 per group, ^***^p < 0.001 bilateral Student's *t*-test. **(B)** Confocal micrographs of hippocampal sections immunostained for Iba1 from control animals (upper panel), and treated animals (lower panel). Inset: Higher magnification confocal micrograph of an Iba1-immunolabeled cell. **(C)** Histogram of the number of GFAP-expressing astrocytes in the dentate gyrus of mice for each experimental condition. Animals: *n* = 3 per group. **(D)** Confocal micrographs of hippocampal sections immunostained for GFAP from control animals (upper panel), and treated animals (lower panel). Inset: Higher magnification confocal micrograph of a GFAP-immunolabeled cell. Blue: Dapi staining. Scale bars: 100 μm, insets 10 μm. N.S: *p* > 0.05. Each value represents the mean ± SEM.

**Figure 7:**
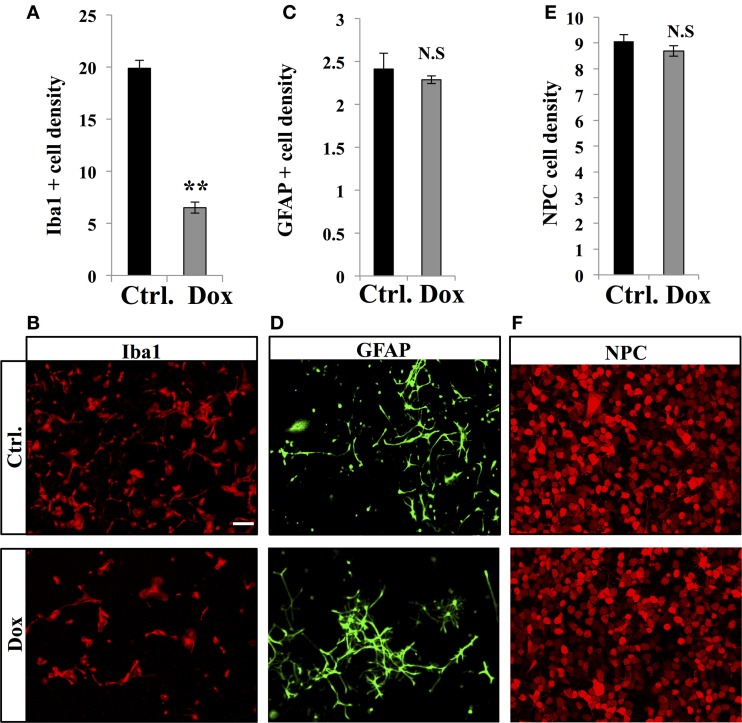
**Effect of Dox on microglia, astrocytes and progenitor cells *in vitro*. (A)** Histogram of the density (×10^−5^ cells/μ m^2^), of Iba1-expressing cells after vehicle (Ctrl.) or Dox treatment of purified microglia cultures. ^**^p < 0.01 bilateral Student's *t*-test. **(B)** Confocal micrographs of Iba1-immunostained cultures for control (upper panel) and treated condition (lower panel). **(C)** Histogram of the density (×10^−4^ cells/μ m^2^), of GFAP-expressing cells of purified cultures treated for each condition. **(D)** Confocal micrographs of GFAP-immunostained cells for control (upper panel) and treated condition (lower panel). **(E)** Histogram of the density (×10^−3^ cells/μ m^2^), of purified NPC for each condition. **(F)** Confocal micrographs of RFP-expressing NPC for control (upper panel) and treated condition (lower panel). *n* = 3 culture wells per group. Scale bar: 100 μm. N.S: *p* > 0.05. Each value represents the mean ± SEM.

Dox is a member of the tetracycline antibiotics group and its analog, minocycline, has been reported to decrease microglia and inhibit microglia activation (Ng et al., 2012). We therefore analyzed the effect of Dox on microglia, identified with immunostaining for the microglia-specific marker Iba1. Four weeks of Dox treatment significantly decreased the number of Iba1-expressing cells in the hippocampus (Figures 6A,B, Student's *t*-test, *p* < 0.001). In contrast, the number of astrocytes, identified with immunohistochemistry for GFAP, was not affected by Dox treatment (Figures 6C,D, Student's *t*-test, *p* = 0.59), indicating that the effect of Dox was specific to microglia. To test the effect of Dox directly on microglia, we performed *in vitro* experiments on purified cell cultures. Microglia was treated with either 1 μg/ml Dox or the equivalent volume of PBS 0.1 M. After 8 days, cells were fixed and immunostained for Iba1. Dox treatment reduced the density of microglia (Figures 7A,B, Student's *t*-test, *p* < 0.01). In contrast, the same treatment did not have any effect on purified astrocytes identified with GFAP immunostaining (Figures 7C,D, Student's *t*-test, *p* = 0.2) or on NPC (Figures 7E,F, Student's *t*-test, *p* = 0.26). Thus, Dox treatment reduced microglia both *in vivo* and *in vitro*.

## Discussion

In this study, we tested the effect on adult neurogenesis of a Dox treatment commonly used to regulate gene expression in inducible transgenic mice. Dox reduced the number of RGL cells but increased the number of TAPs in the DG of adult mice. This may result from a shift in fate choice of the RGL cells leading to a depletion of the RGL cells pool in favor of the highly proliferative TAPs (Encinas et al., 2011), a possibility which may be further tested by examining the expression of the proliferation marker Ki-67 and self-renewal transcription factor sox2 in RGL cells of both groups. As a consequence, Dox treatment increased cell proliferation and resulted in a net increase in neurogenesis. Although cell counts were performed without the use of guard zones, which may lead to possible bias in the counting of cells at the edge of each section, this bias was spread across both groups and may affect the absolute number of cells, but not the intergroup difference. In addition, Dox increased dendritic protrusions density on the neurons formed during the treatment. Concomitantly, microglia but not astrocytes were reduced by Dox. These results indicate that Dox has multiple effects on adult neurogenesis.

Although the mechanism of action of Dox is unclear, the dramatic reduction of microglia *in vitro* and *in vivo*, and the lack of effect of Dox on astrocytes and NPC *in vitro*, raises the possibility that the effect of this drug on adult neurogenesis may be mediated by microglia. In support of this possibility, inflammatory microglia inhibit adult neurogenesis (Monje et al., 2002; Ekdahl et al., 2003; Liu et al., 2007; Kohman et al., 2012) and the exercise-induced increase and the age-dependent decline in neurogenesis are mediated by microglia, possibly through CX3CR1-receptor activity (Vukovic et al., 2012). Furthermore, Dox and its analog minocycline have been shown to inhibit inflammation in models of ischemia, brain trauma, pain or neurodegenerative diseases (Yrjanheikki et al., 1998; Thomas et al., 2003; Buller et al., 2009; Kim and Suh, 2009; Jantzie and Todd, 2010; Lu et al., 2010; Wideroe et al., 2012; Kobayashi et al., 2013). Accordingly, minocycline decrease microglia number and increase adult neurogenesis (Kohman et al., 2013). Thus, an extensive reduction in microglia, such as observed with Dox treatment, may interfere with adult neurogenesis. However, the effects of Dox on microglia and neurogenesis may be unrelated and further experiments will be necessary to examine the causality between reduced microglia and increased neurogenesis upon Dox treatment.

In addition to its effect on microglia, Dox is known to also inhibit Matrix Metalloproteinases (MMPs) (Burggraf et al., 2007; Lee et al., 2009). MMPs are soluble or membrane-bound proteases capable of degrading cell-surface receptors and extracellular matrix proteins, which play a role in tissue remodeling and receptor activation. MMPs are involved in several aspects of neurogenesis: MMP-17 prevents neurogenesis by shedding the ligand for, and activating EGFR (Epidermal growth factor receptor) and its inhibition results in increased neurogenesis (Romero-Grimaldi et al., 2011). MMP-3 and MMP-9 promote the differentiation and migration of neural progenitors (Barkho et al., 2008). Also, MMPs are involved in synaptic plasticity (Bozdagi et al., 2007) and synaptogenesis (Ethell and Ethell, 2007) by the cleavage of trans-synaptic adhesion molecules (Huntley, 2012; Peixoto et al., 2012) and the inhibition of MMP-9 inhibits the LTP-induced spine enlargement and stabilization (Wang et al., 2008). Finally, MMPs are involved in inflammation and the genetic deletion of MMP-9 results in decreased inflammation and neuroprotection in a mouse model of hypoxia (Svedin et al., 2007). Thus, the inhibition of MMPs by Dox may result in increased neurogenesis. However, the involvement of MMPs or microglia on the Dox-mediated increase in neurogenesis remains unclear and additional experiments may elucidate the mode of action of Dox.

Together, our observations indicate that Dox decreased microglia and increased adult neurogenesis, a combination of effects that can introduce confounding factors in studies of adult neurogenesis or brain inflammation. Together with the recent observation that Dox reduced alcohol consumption in mice and increased the sensitivity to alcohol-induced motor impairment (McIver et al., 2012), the present study suggests that, at doses necessary for the induction of gene expression in transgenic mice with tetracycline-responsive promoters, Dox has multiple effects on the central nervous system. Further experiments will be needed to determine whether shorter Dox treatments or other routes of administration, such as intraperitoneal injections, or administration in water induce similar effects on adult neurogenesis and microglia. However, the best experimental design to circumvent the caveats of using tetracycline-responsive promoters relies on the appropriate use of Tet-on or Tet-off systems to enable the experimental tests to be performed in absence of Dox. Alternatively, it is recommended that control experiments include Dox-treated animals. Gene expression control is a critical and increasingly used tool for our understanding of brain physiology and future developments will enable the use of this technology with more specific approaches, thereby reducing confounding factors. In this perspective, the recent introduction of new Tet activators with enhanced sensitivity to Dox represents an interesting effort aimed at using lower doses of Dox, thereby reducing effects on brain physiology (Zhou et al., 2006).

## Author contributions

Conceived and designed the experiments: Sebastien Sultan, Elias Gebara, and Nicolas Toni. Performed the experiments: Elias Gebara, Sebastien Sultan. Analyzed the data: Sebastien Sultan and Elias Gebara. Wrote the paper: Sebastien Sultan, Elias Gebara, and Nicolas Toni.

### Conflict of interest statement

The authors declare that the research was conducted in the absence of any commercial or financial relationships that could be construed as a potential conflict of interest.
